# Global Population Structure of Apple Mosaic Virus (ApMV, Genus *Ilarvirus*)

**DOI:** 10.3390/v15061221

**Published:** 2023-05-23

**Authors:** Ali Çelik, Ali Ferhan Morca, Sevgi Coşkan, Adyatma Irawan Santosa

**Affiliations:** 1 Department of Plant Protection, Faculty of Agriculture, Bolu Abant İzzet Baysal University, 14030 Bolu, Türkiye; 2 Scientifical Industrial and Technological Application and Research Center, Bolu Abant İzzet Baysal University, 14030 Bolu, Türkiye; 3 Directorate of Central Plant Protection Research Institute, Gayret Mah. Fatih Sultan Mehmet Bulv., Yenimahalle, 06172 Ankara, Türkiye; 4 Department of Plant Protection, Faculty of Agriculture, Universitas Gadjah Mada, Jl. Flora No. 1, Sleman, Yogyakarta 55281, Indonesia

**Keywords:** gene flow, phylogenetic study, population genetics, recombination analysis, selection pressure, tripartite genome

## Abstract

The gene sequence data for apple mosaic virus (ApMV) in NCBI GenBank were analyzed to determine the phylogeny and population structure of the virus at a global level. The phylogenies of the movement protein (MP) and coat protein (CP) genes, encoded by RNA3, were shown to be identical and consisted of three lineages but did not closely correlate with those of P1 and P2, suggesting the presence of recombinant isolates. Recombination Detection Program (RDP v.4.56) detected significant recombination signal in the P1 region of K75R1 (KY883318) and Apple (HE574162) and the P2 region of Apple (HE574163) and CITH GD (MN822138). Observation on several diversity parameters suggested that the isolates in group 3 had higher divergence among them, compared to isolates in groups 1 and 2. The neutrality tests assigned positive values to P1, indicating that only this region experiencing balanced or contracting selection. Comparisons of the three phylogroups demonstrated high Fixation index (*F*_ST_) values and confirmed genetic separation and the lack of gene flow among them. Additionally, ±500 bp of partial MP + ‘intergenic region’ + partial CP coding regions of two Turkish isolates from apple and seven from hazelnut were sequenced and determined that their phylogenetic positions fell within group 1 and 3, respectively.

## 1. Introduction

Various herbaceous plants such as hop (*Humulus lupulus*), rose (*Rosa* spp.), and strawberry (*Fragaria* × *ananassa*) and woody plants such as apple (*Malus* spp.), pear (*Pyrus communis* L.), plum (*Prunus domestica*), hazelnut (*Corylus avellana* L.), cherry (*Prunus avium*), peach (*Prunus persica*), apricot (*Prunus armeniaca*), and lichens worldwide were naturally infected by apple mosaic virus (ApMV, genus *Ilarvirus*, Family Bromoviridae) [1,2,3,4,5,6,7]. There is no known vector of ApMV [8], but it is probably transmitted through seed and pollen in hazelnut [9]. The virus provides epidemiological challenge as it also persists in the vegetatively propagated material and is readily graft transmissible [10]. While the virus possibly remains latent in the host, the plants associated with ApMV infection exhibit different types of symptoms including vein banding of leaves, yellow discolorations, and oak-leaf patterns [11].

The virus has a tripartite, positive sense, single stranded RNA genome consisting of RNA-1 (3.4 kb in size), RNA-2 (2.9 kb), and RNA-3 (2.0 kb) [12,13]. Non-structural P1 and P2 proteins that include a methyltransferase domain (MT) and polymerase domain (RdRP) involved in viral replication are encoded by RNA1 and RNA2, respectively. Coat protein (CP), expressed via sub-genomic RNA4, and movement protein (MP) are encoded by the bicistronic RNA3 [14,15].

The genetic variation of ApMV has mostly been investigated by the analysis of the CP gene of isolates from various hosts and geographic locations. Initial analysis of the CP gene of ApMV isolates from Korea revealed three evolutionary groupings distinguished by plant hosts [16]. However, later studies on ApMV isolate sequences from diverse geographical origins throughout the world suggested the existence of two to five evolutionary groups. Seven Indian isolates formed a distinct group separated from the other four groups [2]. Apple and rose isolates from Poland and Belarus were positioned together in one of three constructed phylogroups [5]. These older data maintained some correlation of phylogroups and isolate origins, but perhaps due to frequent global trading, recent reports found no clear association of phylogroups with hosts or locations [6,17,18,19]. Despite this knowledge, detailed diversity and evolutionary analyses involving all regions of the ApMV genome has never been performed, making it difficult to have a general agreement on the exact nature of the virus phylogeny and evolution.

Clear understanding on the population structure of a plant virus could be the key for its accurate detection and effective control [20,21]. Therefore, genome sequences of all global ApMV isolates in the National Center for Biotechnology Information (NCBI) GenBank were analyzed in this study to advance our understanding of the virus at the molecular level. Additionally, Turkey’s 684,000 tons of hazelnut production [22] accounts for 69% of the total world production. The country is also the leader of hazelnut export with a world market share of 61%, followed by Italy, the USA, and Azerbaijan. The presence of ApMV in different host species planted in different regions of the country needs to be molecularly identified as a first step for disease control since considerable yield reduction, up to 42%, had been observed in hazelnut [23,24]. Thus, this study could also clarify the position of known Turkish isolates among global populations.

## 2. Materials and Methods

### 2.1. Alignment Datasets and Recombination Analysis

On 7 February 2023, 9, 5, 20, and 114 isolates (Supplementary Table S1) with complete P1 gene sequence (spans nts 79–3219 in RNA1; reference isolate: accession no. NC_003464), P2 (spans nts 80–2707 in RNA2; reference isolate: accession no. NC_003465), MP (spans nts 169–1029 in RNA3; reference isolate: accession no. NC_003480), and CP (spans nts 1126–1794 in RNA3; reference isolate: accession no. NC_003480), respectively, were retrieved from NCBI GenBank and then aligned according to the respective genome region using Clustal W algorithm suited in MEGA X software v.10.2.4 [25]. Some divergent isolates have different genome lengths with their respective reference isolate due to indel mutations.

Possible recombination signals on the P1, P2, MP, and CP sequences were searched using the RDP, GENECONV, BootScan, MaxChi, ChiMaera, SiScan, and 3Seq algorithms with a Bonferroni-corrected *p* value of <0.05 implemented in Recombination Detection Program (RDP v.4.56) [26]. Only those supported by at least five of the algorithms were included as credible signals.

### 2.2. Phylogenetic and Comparative Nucleotide and Amino Acid Analyses

Four phylogenetic trees based on the P1, P2, MP, and CP genes nucleotide (nt) sequences were built using Neighbor-joining (NJ) algorithms in MEGA X software v.10.2.4. The Kimura 2-parameter models [27] with uniform rate among sites and complete deletion of missing data treatment was determined by the lowest Bayesian information criterion score to be the best for nt substitution of all four alignments. The statistical significance of isolate clusters was determined using 1000 bootstrap replicates. Estimation of the percentage pairwise identities of the P1, P2, MP, and CP coding regions at the nucleotide (nt) and amino acid (aa) levels was calculated using Sequence Demarcation Tool software (SDT v1.2) [28].

### 2.3. Genetic Diversity, Polymorphism, and Neutral Selection Analyses

The diversities on the nt sequences of the P1, P2, MP, and CP genes were evaluated using DnaSP software v.6.12.03 [29] based on the confidence intervals of population genetics-related parameters: the number of haplotypes = *h*, haplotype diversity = *Hd*, average nt differences between sequences = *k*, nt diversity (per site) = π, total number of mutations = η, and the number of variable sites = *S*. Transcriptional selection was determined using a ratio of nonsynonymous to synonymous sites (ω = dN/dS). Neutral selection tests implemented in DnaSP v.6.12.03: Tajima’s *D* [30], and Fu and Li’s D* and F* [31] were performed with a window length of 100 sites and step size of 25 sites to measure genetic divergence on individual coding sequences of the ApMV genome.

### 2.4. Gene Flow and Genetic Differentiation among Populations

The *K*_S_*, *K*_ST_*, Z*, *S*_nn_, and *F*_ST_ values [32,33] were estimated using DnaSP software v.6.12.03. These values determined the genetic differentiation of MP and CP genes nt sequences among compared phylogroups. When genetic divergence among the compared populations is non-existent, the *K*_ST_* value nears zero [34]. A low genetic isolation is translated into a small Z* value [32]. The *S*_nn_ value ranges from a minimum of 0.5 (to show that the compared population was identical) to a maximum of 1 (distinctly separated populations) [33]. The *F*_ST_ value ranges between 0, which is describing strictly identical populations, to 1, which is describing totally distinct populations [32,34]. A high gene flow and large genetic isolation between tested populations are usually indicated by an *F*_ST_ value of at least 0.33 [35,36].

### 2.5. Acquisition of Partial Sequences of Turkish Isolates and Their Phylogeny

Fifteen hazelnut leaf samples from Ordu province and five apple leaf samples from Ankara province, Turkey were collected during the summer of 2022 due to recent reports of viral symptomatic plants in several plantations. Total RNA was extracted from samples using NucleoZOL, one phase RNA purification, following the manufacturer’s procedures (Macherey-Nagel GmbH & Co. KG, Düren, Germany).

Nucleotide sequences of RNA3 of ApMV apple isolates (acc. no. HE574164 (India), KY883320 (Australia), KY971021 (Canada), and MT303103 (Ethiopia)) and hazelnut isolates from Poland (acc. no. HG328282-HG328285) acquired from NCBI GenBank database were aligned using the Clustal W [37] implemented in the MEGA X v.10.2.4 software [25]. The alignment was subjected to Primer-BLAST [38] to generate ApMV-1F (5′-AAGACCCGAAGCCGTAGTTG-3′)/ApMV-1R (5’-GCAAGATCCAGGGTGAGTGT-3′) primers applied in the amplification of a 527 bp of partial MP + ‘intergenic region’ + partial CP coding regions using single step RT-PCR. The 25 μL reaction mixture was composed as follows: 5 µL 5× GoTaq Flexi Buffer, 25 mM MgCl_2_, 10 mM dNTPs, 10 μM each of forward and reverse primer, 200 U Reverse-transcriptase enzyme, 0.2 μL RNase inhibitor (5000 U/mL), 0.25 µL GoTaq polymerase enzyme (5 U/µL), 0.4 µL DMSO, and 2 μL total RNA. The thermal cycler program was as follows: 50 °C for 45 min, 95 °C for 10 min, 35 cycles of 94 °C for 60 s, 62.5 °C for 60 s, and 72 °C for 45 s, followed by a final extension at 72 °C for 7 min. RT-PCR products were loaded onto a 1.5% 1× TAE agarose gel stained with Pronosafe DNA fluorescent marking (Condalab, Madrid, Spain). Electrophoresis was run at 80 Volt for 1 h in a machine (ThermoFisher Scientific, Waltham, MA, USA). The presence of any band was visualized under a UV transilluminator (Vilber, Marne-la-Vall’ee, France). Next, RT-PCR products with the expected band size were sequenced bi-directionally using the Sanger method in BM Laboratory Systems, Turkey.

The recovered nt sequences of Turkish isolates were aligned with those of 14 isolates in NCBI GenBank that have full sequence of RNA3. The alignment was trimmed according to Turkish isolates length. A phylogenetic tree based on the alignment was constructed to investigate their positions in global phylogrouping, using the Neighbor-joining (NJ) algorithms in MEGA X software v.10.2.4 with Kimura 2-parameter as the best supported nt substitution model [27], uniform rate among sites, and complete deletion of missing data treatment. The statistical significance of isolate branching was determined using 1000 bootstrap replicates. Differences in the partial aa sequence of the aligned isolates were examined using BioEdit v.7.2.5 [39].

## 3. Results

### 3.1. Phylogenetic and Recombination Analyses

**Figure 1:**
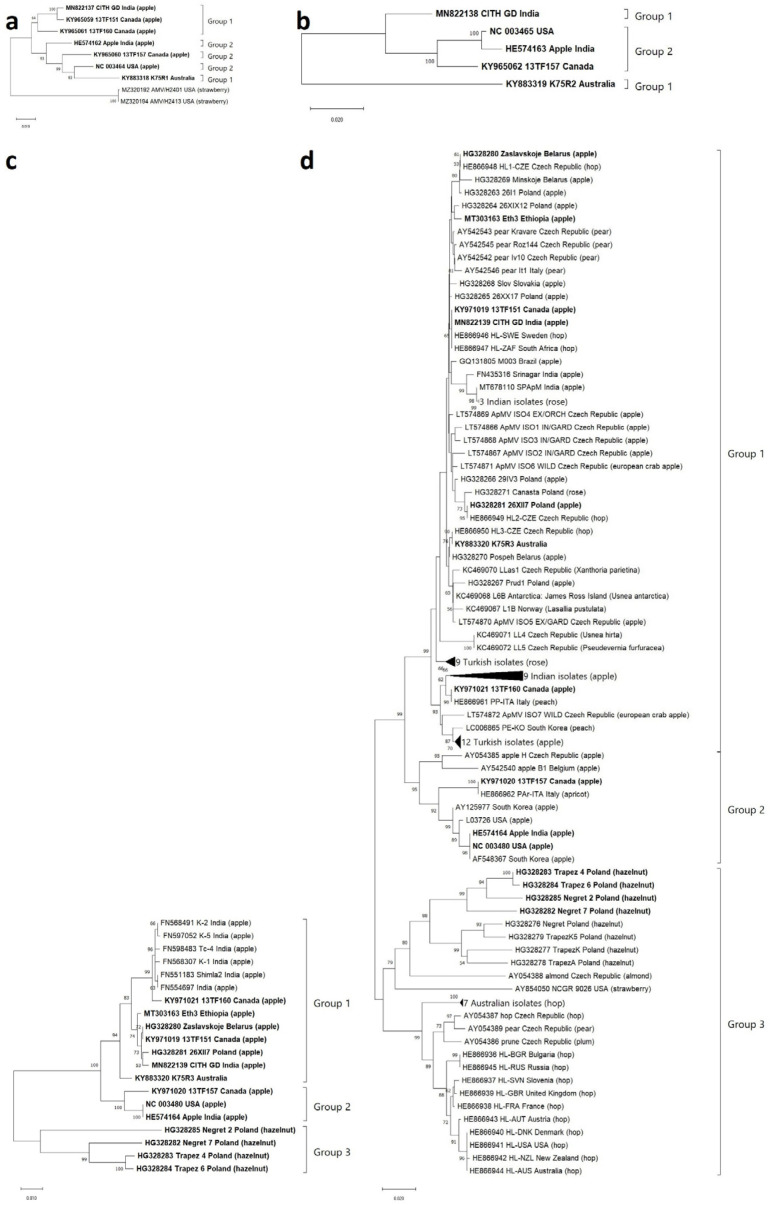
Unrooted phylogenetic trees on the basis of complete nt sequences of four regions of the ApMV genome that were generated by the Neighbor-joining method based on Kimura 2-parameter’s model (1000 bootstrap replicates, only >50% values were shown) with uniform rate among sites and complete deletion of missing data treatment implemented in MEGA X software. Phylogenetic analysis of the complete ORFs of (**a**) P1 gene (3145 nts), (**b**) P2 gene (2634 nts), (**c**) MP gene (862 nts), and (**d**) CP gene (675 nts) are shown. Isolates with a complete RNA3 sequence are printed in bold.

The constructed MP and CP phylogenetic trees showed exactly the same topology, in which global isolates were separated into three main lineages. Fourteen isolates with complete RNA3 sequences were consistently clustered in the same groups in the respective trees (Figure 1; Supplementary Table S1). However, the phylogenies of the P1 and P2 genes could not be resolved in the same way, probably due to recombination events that were found in the P1 region of K75R1 (KY883318) and Apple (HE574162) and the P2 region of Apple (HE574163) and CITH GD (MN822138) (Table 1). Recombinant isolates were removed from subsequent analyses to improve the accuracy of the results.

### 3.2. Comparative Nucleotide and Amino Acid Analyses

According to SDT analysis, the MP and CP genes of ApMV isolates maintained very high nt sequence identities of 88–100% and 87–100%, respectively (Table 2). However, the relatively few nt changes in some isolates (Negret 2 for MP and CP, and TrapezK5, TrapezK, and almond for CP) resulted in aa changes, causing low aa identities of them with those of other tested isolates.

### 3.3. Genetic Diversity and Polymorphism Analyses

Results of the analyses showed that there is a large genomic variation in the sequences of the P1 and P2 genes of few currently known isolates, as shown by the very high *Hd*, *S*, η, and *k* values obtained by both regions. The movement protein gene recorded lower *Hd*, *S*, η, and π values than CP, suggesting that the region is highly conserved when compared to the CP (Table 3). However, there were much less MP sequences available to be compared than the CP sequences.

For both MP and CP, isolates in group 3 exhibited greater diversity among themselves than isolates in group 1 and 2, respectively (Table 3). Therefore, the obtained data could also be an indication that group 3 consists of divergent isolates that might be evolutionary adapted to infect certain hosts, especially hazelnut, since no hazelnut isolate was positioned in the other two groups.

### 3.4. Neutral Selection Analysis

The three parameters of the neutrality test invariably assigned negative numbers, some with statistically significant *p* values, to three phylogroups of the MP and CP genes, which are in RNA3, and gave positive numbers to the P1 gene, which is in RNA1 (Table 4).

### 3.5. Gene Flow and Genetic Differentiation among Populations

Analysis was performed only on the MP and CP genes since there was no clear phylogrouping of the P1 and P2 genes due to a lack of available recombinant free sequences currently listed in NCBI GenBank. The comparisons among groups 1, 2, and 3 of both MP and CP raised high and statistically significant *K*_S_*, *K*_ST_*, Z*, and *S*_nn_ metrics and the Fixation index (*F*_ST_) > 0.33, which thus supported grouping of the constructed phylogenetic trees into three lineages. However, the values presented by *F*_ST_ analysis on the sequences of the MP and CP genes suggested that there were more divergences between group 1 vs. 2 than group 1 vs. 3 and 2 vs. 3 (Table 5).

### 3.6. Phylogeny of Novel Turkish Isolates

**Figure 2:**
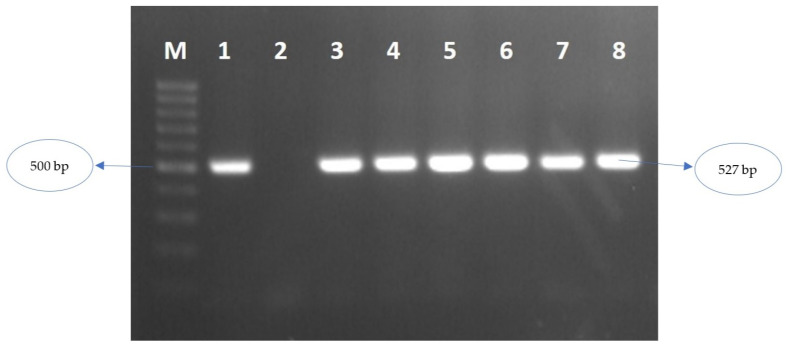
Gel electrophoresis visualization of successful RT-PCR amplification of 527 bp of partial MP + ‘intergenic region’ + partial CP coding regions of new Turkish isolates. **M** ladder; **1** Isolate no. OP374173 from apple; **2** Negative control (uninfected sample); **3** OP374166 from hazelnut; **4** OP374167 from hazelnut; **5** OP374168 from hazelnut; **6** OP374169 from hazelnut; **7** OP374170 from hazelnut; **8** OP374171 from hazelnut.

**Figure 3:**
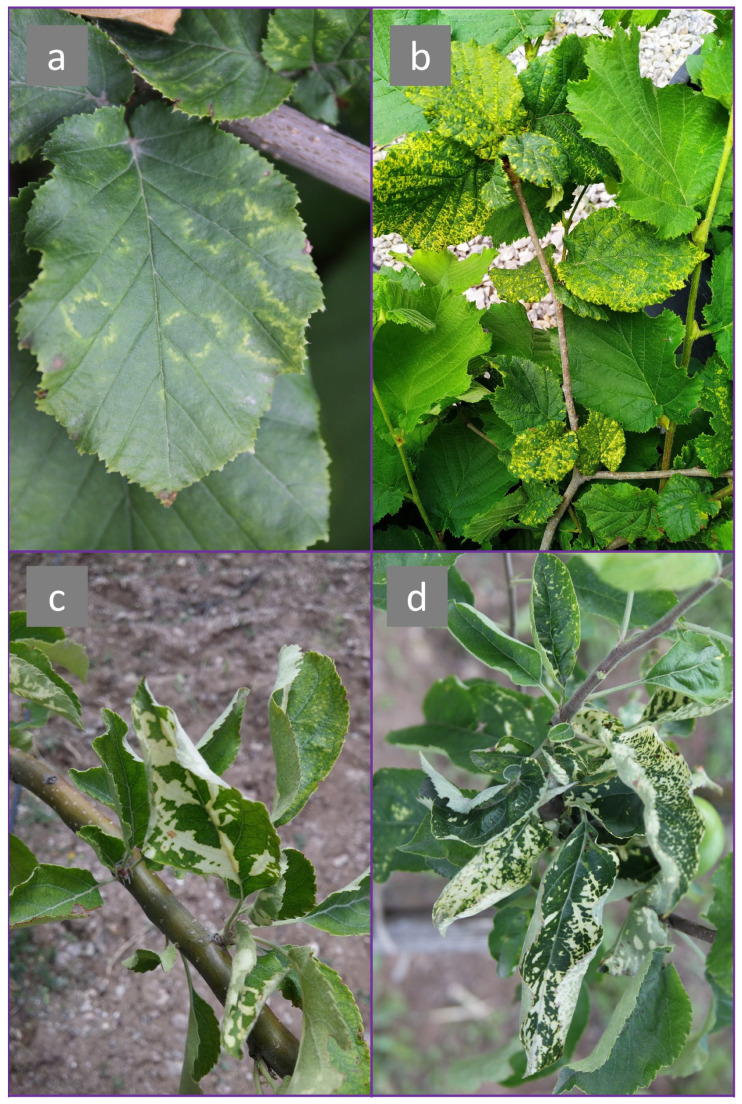
Symptom variation exhibited by ApMV infected plants in Turkey. (**a**) ‘Oak-leaf patterns’ on hazelnut leaves; (**b**) Severe form of ‘oak-leaf patterns’ on hazelnut leaves; (**c**,**d**) White patches on leaves of two different apple plants.

**Figure 4:**
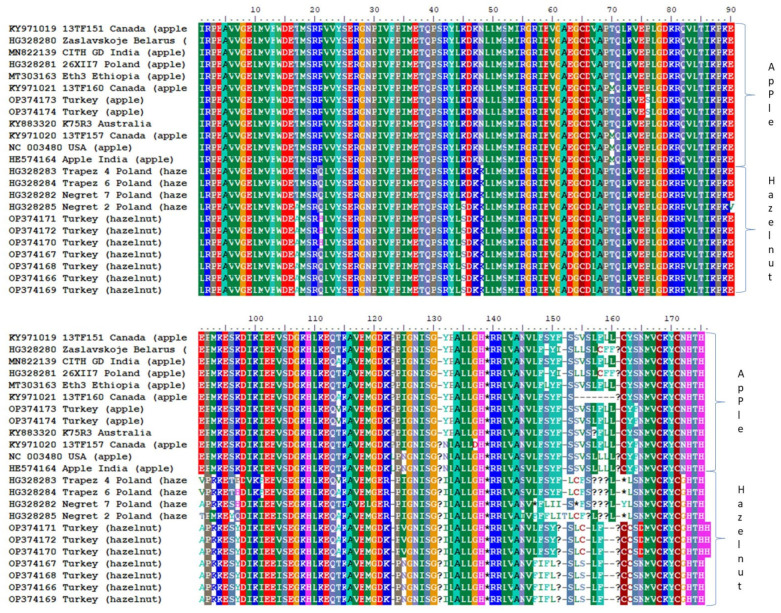
Multiple alignment of the recovered amino acid sequences of partial MP (positions 1–130) + ‘intergenic region’ + partial CP (positions 166–176) genes of genome of Turkish ApMV isolates using different colors to show variation between apple and hazelnut isolates. ApMV RefSeq NC_003480 was included in the alignment.

All of 20 samples were positive for ApMV infection according to results of a molecular test using RT-PCR (Figure 2). Infected hazelnut showed distinctive ‘oak-leaf patterns’ symptoms, whereas infected apple exhibited white discoloration symptoms (Figure 3). Sequencing of nine positive RT-PCR products gave around 500 nts of the ApMV genome, covering partial MP + ‘intergenic region’ + partial CP gene (spans nts 637–1156 in RNA3; reference isolate: accession no. NC_003480). Some Turkish isolates showed different nt and aa lengths due to indel mutations (Figure 4). Newly obtained sequences were registered in NCBI GenBank with accession no. OP374166-OP374174.

**Figure 5:**
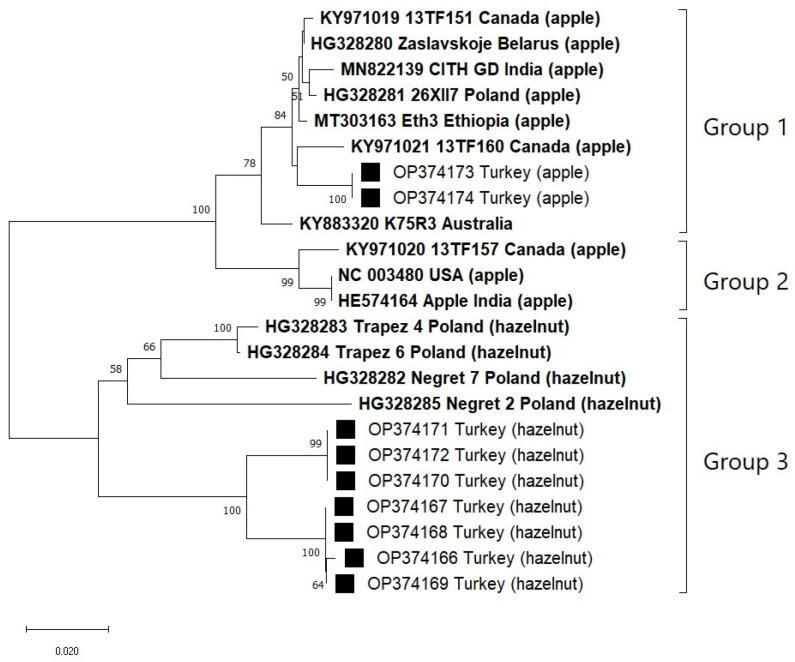
Unrooted phylogram based on ±500 nts of partial MP + ‘intergenic region’ + partial CP genes of genome of nine new ApMV Turkish isolates (highlighted with black squares) and 14 NCBI GenBank isolates with complete RNA3 sequence (printed in bold). The phylogram was generated by the Neighbor-joining method based on Kimura 2-parameter’s model (1000 bootstrap replicates, only >50% values were shown), with uniform rate among sites and complete deletion of missing data treatment implemented in MEGA X software.

The constructed phylogenetic trees positioned two Turkish isolates from apple and six other apple isolates in group 1, whereas seven Turkish isolates from hazelnut were clustered with other four hazelnut isolates identified in Poland in group 3 (Figure 5). Therefore, there was some degree of correlation between ApMV phylogeny with host species. The results were in line with those of polymorphism analysis, which indicated that group 3 belongs to evolutionary successful isolates capable to acquire hazelnut as an additional host. Results of the molecular study also verified the new primer pair capability to amplify partial genome of genetically diverse isolates belong to different hosts and phylogroups.

## 4. Discussion

ApMV is a major concern in stone fruits and other high value crops cultivated around the world, but its complete phylogeny is still unclear. In this current work, all complete sequences of the P1, P2, MP, and CP regions of the ApMV genome currently listed in NCBI GenBank were analyzed to better understand the population structure and evolution course of the virus. ApMV has also been studied quite well in Turkey, and some isolates have been sequenced. However, sequences of hazelnut isolate, which was reported to be genetically divergent [6], are not yet available. Therefore, hazelnut and apple isolates from Ordu and Ankara provinces obtained in this study were partially sequenced and then studied to know their molecular characters.

RDP analysis of this study did not only confirm the recombination in the P2 region of MN822138 recently reported by [11], but also found strong signals in the P2 region of HE574163 and in the P1 region of HE574162 and KY883318. However, a recombination event was not found in the sequences of the MP and CP genes. Phylogenetic trees further showed that while MP and CP shared an exact phylogeny, P1 and P2 did not have the same phylogeny. Given the frequently occurred signals on these few known sequences of P1 and P2, it is likely that recombination plays more important roles in shaping ApMV evolution, particularly in the two regions than reassortment. However, more sequences of P1 and P2 are clearly needed to draw a strong conclusion on this issue.

There was no strong agreement on ApMV phylogeny as most of the phylogenetic trees of the virus were constructed using the Neighbor-joining (NJ) method [2,5,6,18], while at least one applied Maximum-likelihood [17]. Thus, these proposed trees sometimes produced rather distinctive branches. To resolve this, phylogenetic trees constructed in the current study employed the most commonly used NJ method. Furthermore, unrooting the trees from any out-group seemed to increase the ease and accuracy of phylogrouping. As a result, the trees based on the complete sequences of MP and CP regions (both are in RNA3) showed exactly the same topology in which both trees were divided into three main phylogroups (groups 1, 2, and 3) (Figure 1). Prominently, the trees showed no correlation between the groups and hosts and origin, indicating a wide host range and distribution of any ApMV strain. Phylogrouping proposed in this study could be a strong base for future molecular study of ApMV since all GenBank isolates from different hosts and countries were involved in the analysis.

Both MP and CP regions actually maintained relatively high sequence identity among the isolates according to the SDT study. However, changes in the nt sequences of several isolates that were mostly recovered from hazelnut and belong to a subcluster in group 3 (Negret 2 for MP and CP) and TrapezK5, TrapezK, and almond for CP were translated into many non-synonymous substitutions; thus, their aa sequences shared very low identity with other isolates. Out of curiosity, separate polymorphism and neutral selection analyses were performed on the CP sequences of the 10 isolates in this subgroup within group 3, named ‘Hazelnut’ (all the available hazelnut isolates already belong to group 3 in MP comparison). In the CP comparison, the only 10 isolates in the Hazelnut subcluster were shown to have higher variance among them, according to all diversity parameters, and experienced weaker selection pressure (ω = 0.971) than those of the whole members of groups 1 and 2 (Table 3). The Hazelnut subgroup also received negative values from three neutrality tests (Table 4). Therefore, the results further distinguished the divergence of these isolates that adapted to infect hazelnut.

In this study, seven hazelnut isolates from Turkey were partially sequenced to complement our knowledge on the only known hazelnut isolates obtained in Poland [6]. Results of this phylogenetic study showed that even though new hazelnut and apple isolates were collected from nearby provinces within the same country, the Turkish hazelnut isolates were related much closer to other Polish isolates from hazelnut than to Turkish apple isolates. As a matter of fact, the Turkish apple isolates were clustered with other apple isolates from different countries (Figure 5). Similar to the Polish isolates, there were nt deletions in the RNA3 sequence of Turkish hazelnut isolates at positions nts 1003–1005 and 1089–1091 (reference isolate: accession no. NC_003480), which were reflected into deletion of one aa residue in the respective positions at translation of this segment. However, all seven Turkish isolates also experienced unique additional ten nt deletions at positions nts 1098–1100 and 1106–112 that resulted in the further removal of a total of four aa residues (Figure 4). Most of these aa changes at RNA3 between the apple and hazelnut isolates happened at the non-coding intergenic region (Figure 4), thus unlikely to affect isolates fitness.

Overall, all ApMV phylogroups were under weak evolutionary constraint, which could be one of the reasons for high genetic diversity among isolates in all three groups. In line with this finding, it can also be suggested that different ApMV populations, which all were constantly assigned negative neutrality values, are experiencing further expansion (population growth due to lack of subdivision) or bottleneck selections. Interestingly, these results were rather contrary to those experienced by prunus necrotic ringspot virus (PNRSV), another member of *Ilarvirus* that also infects stone fruits worldwide, which was found under very strong purifying pressures and received positive neutrality values for some of its populations [40].

High and statistically significant *K*_S_*, *K*_ST_*, Z*, and S_nn_ metrics obtained by all group comparisons confirmed that the clustering of isolates into three phylogroups in both MP and CP trees has been done correctly. Furthermore, estimation of genetic separateness based on *F*_ST_ values > 0.33 showed that members of these groups were genetically distinct, and there was a lack of gene flow among them.

## 5. Conclusions

The P1, P2, MP, and CP regions in the genome of all the isolates registered in NCBI Genbank were analyzed to study ApMV phylogeny and also to understand the global population structure of the virus. Both MP and CP trees shared the same topology in which isolates were clustered into three major groups (1, 2, and 3) without correlation with host species and countries. Two new Turkish isolates from apple and seven from hazelnut were positioned within group 1 and 3, respectively. However, the clustering cannot confirm P1 and P2 phylogenies yet due to a lack of available data. For MP and CP, calculation on different parameters estimated relatively higher genetic diversity among isolates in group 3 than those in groups 1 and 2. P1 was the only genome region sustaining a balanced or contracting selection. It is clear that additional molecular data are still needed to complete the analysis on the P1 and P2 regions.

## Figures and Tables

**Table 1:** Putative recombination events detected in the P1 and P2 regions by RDP4 analysis of ApMV isolates.

No.	Recombinant	Parents: Major/Minor	Breakpoints ^1^ (Start/End)	RDP Implemented Method ^2^ (*p* Value)
P1				
1.	KY883318 (Australia)	NC_003464 (USA)/unknown	2365/2790	R (6.564 × 10^−21^) G (8.039 × 10^−20^) B (2.271 × 10^−15^) M (1.136 × 10^−15^) C (1.831 × 10^−13^) S (3.149 × 10^−10^) 3S (5.593 × 10^−28^)
2.	KY883318 (Australia)	NC_003464 (USA)/KY965059 (Canada)	360/3062	R (9.084 × 10^−12^) G (1.468 × 10^−7^) B (8.045 × 10^−12^) M (3.028 × 10^−8^) C (5.966 × 10^−7^) S (1.456 × 10^−4^) 3S (2.376 × 10^−6^)
3.	HE574162 (India)	KY965061 (Canada)/NC_003464 (USA)	1288/2063	R (6.740 × 10^−16^) G (3.307 × 10^−14^) B (3.122 × 10^−16^) M (3.925 × 10^−11^) C (1.939 × 10^−11^) S (2.879 × 10^−14^) 3S (9.453 × 10^−16^)
4.	HE574162 (India)	KY965061 (Canada)/unknown	2558/2879	R (2.457 × 10^−3^) G (1.522 × 10^−3^) B (1.570 × 10^−4^) M (6.996 × 10^−4^) C (6.122 × 10^−4^) S (4.158 × 10^−4^) 3S (5.398 × 10^−4^)
P2				
1.	HE574163 (India)	KY965062 (Canada)/unknown	493/1125	R (2.001 × 10^−8^) G (4.360 × 10^−4^) B (4.893 × 10^−5^) M (1.883 × 10^−9^) C (2.128 × 10^−3^) S (3.546 × 10^−6^) 3S (4.883 × 10^−6^)
2.	MN822138 (India)	KY883319 (Australia)/KY965062 (Canada)	1132/2458	R (3.834 × 10^−11^) G (3.646 × 10^−10^) B (3.876 × 10^−9^) M (1.759 × 10^−12^) C (4.075 × 10^−6^) S (1.325 × 10^−39^) 3S (1.322 × 10^−26^)

^1^ Position in alignment; ^2^ R = RDP; G = GENECOV; B = BootScan; M = MaxChi; C = Chimaera; S = Siscan; 3S = 3Seq.

**Table 2:** Nucleotide (nt) and amino acid (aa) identities in the P1, P2, movement (MP), and coat protein (CP) genomic regions among ApMV phylogroups.

Genomic Region	Identity (%)	
	nt	aa
**P1**	91–99	78–99
**P2**	87–96	89–96
**MP**	88–100	69–100
group 1 × group 2	95–97	83–92
group 1 × group 3	88–90	69–75
group 2 × group 3	88–90	70–77
**CP**	87–100	69–100
group 1 × group 2	93–94	77–88
group 1 × group 3	87–91	69–78
group 2 × group 3	87–91	69–78

**Table 3:** Summary of genetic diversity and polymorphism analyses of four ApMV genomic regions from different populations.

Phylogroups	*N*	*h*	*Hd*	*S*	η	*k*	π	dS	dN	ω
**P1**	7	7	1.000	389	407	177.143	0.056	0.092	0.047	0.511
**P2**	3	3	1.000	381	389	256.667	0.112	0.275	0.047	0.171
**MP**	20	17	0.984	174	194	46.663	0.055	0.059	0.053	0.898
Group 1	13	11	0.974	38	38	11.590	0.013	0.018	0.012	0.667
Group 2	3	2	0.667	17	17	11.333	0.013	0.021	0.011	0.524
Group 3 (=Hazenut)	4	4	1.000	102	105	55.333	0.064	0.074	0.062	0.838
**CP**	114	87	0.994	257	321	37.870	0.063	0.073	0.059	0.808
Group 1	75	54	0.990	136	149	17.081	0.027	0.031	0.026	0.839
Group 2	9	6	0.889	63	65	23.583	0.035	0.038	0.032	0.842
Group 3	30	27	0.991	198	235	49.085	0.076	0.094	0.069	0.734
Hazelnut	10	10	1.000	161	183	58.822	0.089	0.101	0.098	0.971

*N*: number of isolates, *h*: number of haplotypes, *Hd*: haplotype diversity, *S*: number of variable sites, η: total number of mutations, *k*: average number of nucleotide differences between sequences, π: nucleotide diversity (per site), dN: non-synonymous nucleotide diversity, dS: synonymous nucleotide diversity, ω: dN/dS.

**Table 4:** Summary of demography test statistics of four ApMV genomic regions from different populations.

Phylogroups	Fu and Li’s *D* *	Fu and Li’s *F* *	Tajima’s *D*
**P1**	0.78257 ns	0.77054 ns	0.39048 ns
**P2**	nd	nd	nd
**MP**	−0.23355 ns	−0.23355 ns	−0.60877 ns
Group 1	−0.94708 ns	−0.86588 ns	−0.23742 ns
Group 2	nd	nd	nd
Group 3 (=Hazelnut)	−0.26300 ns	−0.29969 ns	−0.35436 ns
**CP**	−2.54107 *	−2.34983 *	−1.24742 ns
Group 1	−3.45373 **	−3.1910 **	−1.50761 ns
Group 2	0.29956 ns	0.23542 ns	−0.07131 ns
Group 3	−0.98120 ns	−1.03740 ns	−0.66893 ns
Hazelnut	−0.44376 ns	−0.50406 ns	−0.45212 ns

* 0.01 < *p* value < 0.05; ** 0.001 < *p* value < 0.02, ns = not significant, nd = not determined, at least four isolates are needed for analysis.

**Table 5:** Genetic differentiation estimates for lineages of ApMV, based on sequences of MP and CP genomic regions comparisons.

Comparison	^α^*K*_S_ *	^α^*K*_ST_ *	*p* Value	^α^*Z* *	*p* Value	*S*_nn_	*p* Value	^β^ *F*_ST_
MP								
Group 1 (n = 13)/Group 2 (n = 3)	2.2753	0.1651	0.0020 **	3.4211	0.0010 **	1.0000	0.0020 **	0.6874
Group 1 (n = 13)/Group 3 (n = 4)	2.5339	0.2197	0.0000 ***	3.5449	0.0000 ***	1.0000	0.0000 ***	0.6591
Group 2 (n = 3)/Group 3 (n = 4)	3.1513	0.2026	0.0070 **	1.4256	0.0330 *	1.0000	0.0330 *	0.6493
**CP**								
Group 1 (n = 75)/Group 2 (n = 9)	2.7341	0.0677	0.0000 ***	6.9405	0.0000 ***	1.0000	0.0000 ***	0.5428
Group 1 (n = 75)/Group 3 (n = 30)	2.9091	0.1289	0.0000 ***	7.2177	0.0000 ***	0.9905	0.0000 ***	0.5093
Group 2 (n = 9)/Group 3 (n = 30)	2.7341	0.0677	0.0000 ***	6.9405	0.0000 ***	1.0000	0.0000 ***	0.5428

* 0.01 < *p* value > 0.05; ** 0.001 < *p* value > 0.01; *** *p* value < 0.001; ^α^*K*_S_*, *K*_ST_*, Z* and *S*_nn_ are test statistics of genetic differentiation; ^β^*F*_ST_, coefficient of gene differentiation, which measures inter-population diversity.

## Data Availability

The new data presented in this study are openly available in the NCBI GenBank database, with accession numbers OP374166-OP374174.

## Supplementary Materials

The following supporting information can be downloaded at: https://www.mdpi.com/article/10.3390/v15061221/s1, Supplementary Table S1: ApMV isolates tested in this study.
